# Immunotherapy generates selective pressure for acquisition of immunogenic neoantigens in escape tumors

**DOI:** 10.1186/2051-1426-3-S2-P68

**Published:** 2015-11-04

**Authors:** Jenny H Pan, Suman Vodnala, Robert L Eil, Zhiya Yu, Jared J Gartner, David Clever, Rahul Roychoudhuri, Shashank J Patel, Christopher A Klebanoff, Madhusudhanan Sukumar, Tori Yamamoto, Nicholas P Restifo

**Affiliations:** 1 Surgery Branch, National Cancer Institute, National Institutes of Health, Bethesda, MD, USA; 2 Center for Cancer Research, NCI/NIH, Bethesda, MD, USA

## 

Host immunosurveillance as a mechanism to promote tumor growth or to suppress tumor development is well studied. It is less known how immuno-selective pressure in the form of immunotherapies can instruct the immune system during this process. We hypothesized that the application of a T cell-dependent selection pressure in the form of a whole tumor vaccine would alter the immunogenic architecture in our transplantable murine melanoma model SB-3123, and generated an escape variant to test this hypothesis.

We performed whole-exome and RNA-sequencing of the tumor line SB-3123 and two fresh tumors generated by subcutaneous implantation of SB-3123 to identify mutations and to assess levels of expression. There were 349 mutations shared between the tumor line and fresh tumors, with less than 5 mutations that were unique to each sample. We then determined the stability of these mutations and created a re-derived tissue culture line from a fresh SB-3123 tumor. Remarkably, the re-derived tissue culture line retained 333 mutations in common with the prior samples and produced only 2 unique mutations.

To test the immunogenicity of SB-3123, we vaccinated mice with irradiated SB-3123 and administered a live tumor challenge two weeks after vaccination. This resulted in a profound vaccination response and mice were completely protected from tumor for over 200 days. Surprisingly, one vaccinated mouse developed a recrudesced tumor at the site of implantation 40 days after challenge, and a tumor line was created (SB-3123-esc). We asked whether SB-3123-esc represented an antigen escape variant by immunizing mice with SB-3123 and challenging with either SB-3123 or SB-3123-esc. Vaccination with SB-3123 did not protect against SB-3123-esc. We also performed sequencing on SB-3123-esc and determined that no known genes involved in antigen processing and presentation were mutated, and SB-3123-esc upregulated class I molecules at similar levels compared to SB-3123 upon IFN-γ treatment.

Our most striking results were the differences in mutational constituents between SB-3123 and SB-3123-esc. Of the nonsynonymous mutations called in the coding region, 441 were common to both tumor lines while 80 were unique to SB-3123 and 40 to SB-3123-esc. Furthermore, we were able to determine that SB-3123-esc lost 2 immunogenic neoantigens but had acquired 5. Future investigations are directed towards identifying the rejection antigen responsible for the escape phenotype of SB-3123-esc.

